# A Commentary on Miron et al. (2024): Why Managers May Not Actually Stop Caring About Gender Inequality

**DOI:** 10.1177/10596011241312453

**Published:** 2025-01-06

**Authors:** Christine C. Hwang, Laurie J. Barclay, Marion Fortin, Michael R. Bashshur, Ann Pegoraro

**Affiliations:** 1 3653 University of Guelph, Guelph, Canada; 2 27089 Toulouse 1 University Capitole, Toulouse, France; 3 54756 Singapore Management University, Singapore

**Keywords:** organizational justice, gender, diversity, gender diversity, motivated cognition

Drawing upon the Motivated Perceptual Approach (MPA; Bashshur et al., 2023), we challenge Miron et al.’s (2024) overall conclusion that managers “stop caring about organizational justice”. We first analyze Miron et al. (2024) to critique the overgeneralization of the scholarly assumptions and conceptualizations and also raise concerns about the ecological validity of the findings. We then showcase how applying the MPA to the questions raised by Miron et al. (2024) can enhance theoretical and practical insights to advance gender equity, including providing actionable practical strategies people can use to bring inequality to others’ attention. Taken together, we encourage scholars and practitioners to share information that can prompt others to perceive and respond to inequality as well as enhance equality in organizations. Given persistent gender inequalities in the workplace, Miron et al. (2024) question whether managers’ power prompts them to “stop caring” about gender inequality. We appreciate the spotlight this article places on recognizing that gender inequality “should be treated as an organizational justice issue” (p. 27). We also agree that it is critical to identify what prevents gender inequality from being perceived and addressed. However, we disagree with the overall conclusion that “with increased power, managers stop caring about organizational justice” (p. 26). Beyond concerns with the validity of the conclusion, we are also concerned that this framing may discourage people from taking steps that are within their control to promote gender equality.

In this commentary, we challenge key assumptions within Miron et al.’s theorizing and apply the *Motivated Perceptual Approach* (MPA; Bashshur et al., 2023) to question the validity of their overall conclusion. By doing so, we illustrate that managers do not necessarily “stop caring” but rather may have perceptual differences and/or constraints in their ability to act. We begin by overviewing the MPA, then critically analyze Miron et al.’s assumptions and outline how replacing those with the MPA can further enhance theoretical and practical insights.

## What is the Motivated Perceptual Approach (MPA)?

The Motivated Perceptual Approach (MPA) is grounded on the foundational notion that fairness perceptions are subjective because people are “motivated to selectively perceive and weight cues [information] to form fairness perceptions that align with their motives” (Bashshur et al., 2023, p. 1). As such, people may perceive the same situation differently because they may not have access to the same cues and/or may be differentially motivated to attend to, select, evaluate, or weight cues. People can also socially construct and negotiate perceptions by sharing information to expand others’ access to relevant cues, provide information related to the importance and credibility of cues, create a shared “cue set” to find common ground, and/or activate different motives to influence the specific cues that others are likely to select, evaluate, and/or weight.

### Beyond Standards of Injustice: The Importance of Expanding the Cue Set

Miron et al. focus on standards of injustice as a singular cue that managers rely on to inform perceptions of gender inequality and argue that those in power have higher standards of injustice, such that the “amount of evidence needed to conclude that workplace gender wage inequality is unfair to female employees” is increased (p. 2). Further, Miron et al. equate the tendency to endorse a higher threshold to a *lack of care about justice*. We argue that focusing on a *single* cue and *this* particular cue is problematic for two key reasons.

First, *standards of injustice are unlikely to be the only cue that inform people’s subjective judgements and/or behavioral reactions to gender inequality, and its degree of relevance is likely to vary*. Standards of injustice are broadly operationalized as the percentage of (a) occupations that have a wage discrepancy, (b) women who are economically disadvantaged by a wage discrepancy, and (c) men who are earning salaries that are more than women (across a lifetime). However, simply because a wage discrepancy exists does not mean it is *unfair* (e.g., Brockner, 2011). For example, differences in occupational demands or work arrangements may create disparities. Fair processes can also result in unfavorable outcomes (and managers typically have access to cues about the fairness of the process). When unfair discrepancies exist, focusing on the *degree* of discrepancy may not capture people’s perceptions (e.g., those who believe that *all* people should be treated fairly may perceive even small discrepancies as highly unfair; Bies, 2001). Moreover, managers are unlikely to have complete control over broad wage discrepancies and gender inequality goes far beyond wage discrepancies. This raises questions about whether Miron et al.’s approach suffers from construct deficiency and whether (and to what degree) managers are likely to perceive this cue as relevant and use it to inform their *own* perceptions.

Second, *equating standards of injustice to ‘justice’ falls into the trap of reification*. Reification arises when an operationalization is treated “as though it represents the *actual* phenomenon” and captures a “perfect representation” of the construct (Rupp et al., 2017, p. 940). This threatens validity by failing to specify the assumptions underlying an operationalization. Beyond the embedded assumptions noted above, Miron et al.’s interpretations overlook that scholarly conceptualizations may not reflect people’s lived experiences (Rupp et al., 2017). Also, within the literature, justice and injustice have been argued to be distinct constructs with disparate processes (Goldman & Cropanzano, 2015). As such, equating standards of *injustice* with *justice* may create conceptual ambiguity and prevent a clear sense of how inequality can be addressed *and* equality can be fostered. Taken together, we are concerned about the validity of Miron et al.’s interpretations, especially since evidence is not provided that abstract statements about wage discrepancies sufficiently capture overall perceptions of gender inequality, managers stop caring about *justice*, and/or broad assessments related to standards of injustice across occupations are influential for managers’ perceptions and behaviors in their *own* organizations.

Whereas Miron et al. focus on a singular cue that reflects an absolute judgement, the MPA is grounded on the notion that perceptions are subjective and may incorporate a diverse set of cues. Indeed, voluminous research has shown that justice perceptions can encapsulate a wide range of information, including diverse sets of justice standards (e.g., German et al., 2016), and multiple cues can be selected, evaluated, and weighted (e.g., Bashshur et al., 2023). Applying the MPA lens to Miron et al.’s arguments draws attention to the importance of more fully examining the extent to which people use standards of injustice to inform their justice perceptions (e.g., select this as a relevant cue), how they evaluate this cue (e.g., whether people do indeed focus on the quantity rather than *credibility* of the evidence; see Kunda, 1990), and how this cue may be weighted against other cues to inform perceptions, especially since managers typically have access to a broad and rich set of contextualized cues that non-managers may not.

Building on this foundation, women (vs. men) may have “lower thresholds” for deeming inequality as unfair because they are more likely to have personal experiences with gender inequality and its negative implications (have differential access to, select, evaluate, and weight cues related to gender inequality). Similarly, managers may have “higher thresholds” for deeming gender inequality as a systemic issue for several reasons. For instance, their role and responsibilities provide access to *different* cues (which may call into question whether disparities are unfair). Further, managers may need to defend their assessment that gender inequality exists *and* is unfair. This is a key point because managers “can believe what they want but only to the extent that reason, cognitive capacity, and the available information permits” (Bashshur et al., 2023, p. 6). Rather than assuming that managers “stop caring about organizational justice”, they may instead be experiencing a “credibility bias” (see Pornpitakpan, 2004) in which they may need more evidence to feel like they are making a defensible judgement. Moreover, managers may still care about gender inequality but may not have the discretion to act when it is deemed to be a systemic issue. This may prompt hesitancy to label it as a systemic issue or focus managers on addressing gender inequality in other ways that are under their discretion or purview.

### Broadening and Exploring the Intersection of Motives to Enhance Ecological Validity

Miron et al. argue that managers are motivated to protect their identity (identity motive), their own resources (self-interest motive), and/or the status quo (system justification motive), which results in managers raising the standard of injustice (and not caring about justice). While we agree that multiple motives can be simultaneously activated, we are concerned about the assumptions underlying this argument and the resulting interpretation of the findings.

First, *we challenge the notion that the activation of an identity motive equates to caring less about justice*. While managers can be motivated to see their organization as equitable (e.g., Ellemers & van den Bos, 2012) and are less likely to perceive systemic inequality within their own organizations (e.g., To et al., 2022), highly identified managers can be *more* motivated to address overt acts of discrimination within their organization (e.g., Gloor et al., 2024). Further, identity motives are not limited to diminishing identity threats but may also focus managers on pursuing opportunities (e.g., cultivating and maintaining a positive self-image by addressing inequality and fostering equality; Scott et al., 2014). Taken together, while managers may have difficulty perceiving inequality, the activation of identity motives can encourage them to foster equality and address inequality when it is perceived.

Second, *we challenge the argument that primarily women are motivated to advance gender equality and that managers are motivated to maintain inequities.* Extensive research has shown that men also care about gender equality and the benefits of gender equality are not limited to women but extend to all genders while also benefiting organizations (e.g., Fine et al., 2020). As Kofi Annan, former secretary general of the United Nations, eloquently noted, “Gender equality is not a woman’s issue, it is a human issue. It affects us all.” As such, we question the validity of assuming that men are primarily interested in pursuing their own self-interest. Indeed, as a justice issue, inequality may also activate moral motives (Barclay et al., 2017). Moreover, Miron et al.’s arguments imply that managerial and gender identities are mutually exclusive and/or managerial identity overrides gender identity. However, people often navigate tensions and/or share meanings across identities (e.g., Thrasher et al., 2024). For instance, beyond recognizing that the onus should not be on women to advance gender equality, managers may also perceive that their responsibilities *as a manager* include promoting gender equality.

Third, *we challenge the notion that identity motives are the primary and/or dominant motives that are activated.* By virtue of being responsible for and/or interacting with their employees, managers can be motivated to fulfill the moral duties associated with their role (moral motive) or want to protect their relationships with employees (relational motive). This simultaneous activation of multiple motives can attune managers to perceive injustice that their employees are experiencing *and* motivate them to address it (e.g., due to the implications for themselves *and* their employees). Moreover, this can focus attention on cues that go beyond standards of injustice (a relatively abstract and generalized cue) to those cues that are relevant to the specific situation in their organization. This raises concerns about the ecological validity of the findings (i.e., whether the findings are generalizable to real-world settings and interactions).

Applied to Miron et al.’s theorizing, the MPA highlights the importance of considering that multiple motives may be activated and that people’s role(s) in the situation may influence which motives are activated. Further, the interaction of these motives may influence which cues are available as well as how people select, evaluate, and weight the cues. Identifying which motives are most likely to be activated in a situation and how these motives can influence the cue set (individually and in tandem) is important because the various motives can reflect different psychological needs, drive distinct behaviors, and require disparate interventions. For example, a self-interest motive may be activated by anxiety, focus managers on behaviors that benefit themselves, and be attenuated with prosocial messages that broaden attentional focus to include others (e.g., Hillebrandt & Barclay, 2023). By contrast, system justification motives may arise from psychological needs for coherence (e.g., Jost & van der Toorn, 2012), focus managers on rationalizing inequality by delegitimizing those who experience disadvantage (e.g., Lyubykh et al., 2024), and be mitigated by activating system change motives (e.g., Lyubykh et al., 2022).

Whereas “motives matter” because these can impact the way that managers perceive and respond to inequality, we also argue that “context matters” because this can influence which motives are activated as well as amplify or attenuate their impact. For instance, Arvate et al. (2018) challenged the notion that women in positions of power undermine the advancement of other women (behave as “queen bees”). Instead, their findings show that when women leaders have decision-making discretion, the numbers of women in top and middle level management increase in those organizations. This suggests that it is not a dearth of justice motives for women in power but rather a *context* that constrains or amplifies the ability to act on them. Managers may also receive signals from their broader environment that can impact their perceptions and/or behavior within the organization (e.g., Hillebrandt & Barclay, 2023). For example, in the wake of the 2024 US election, social media was flooded with misogynistic comments that normalized gender inequality (e.g., “your body, my choice”; Duffy, 2024). This may impact which motives are activated and normalize gender inequality, thereby making some American managers less prone to perceive or react to gender inequalities while attuning others to gender inequalities (e.g., women, employees and managers in multi-national branches of a US company).

## Practical Insights From the MPA to Promote Gender Equality

We are also concerned that the conclusion that managers “stop caring about justice” may disempower those that have the potential to make change on an everyday basis in the workplace (e.g., Kiefer et al., 2024). For example, this may dissuade managers from engaging in allyship, collectively working towards gender equality, and/or making everyday changes – changes that can ultimately create and support momentum for broader social changes.

To empower managers, we suggest that it is important to expand our focus to what *can* be done, including what facilitators can be leveraged and how potential barriers can be overcome to support gender equality (see Bashshur et al., 2023; Lyubykh et al., 2022 for additional strategies and interventions). For example, managers may benefit from gathering information to ensure that relevant, important, and informative cues have been identified. Further, asking managers to outline their rationale can activate accuracy motives (Kunda, 1990), thereby encouraging managers to consider and critically evaluate the full set of cues to arrive at an informed (and justifiable) judgment. Organizations may also benefit from sending signals that they are committed to improving gender equality (e.g., messaging that the organization is committed to continuous improvement). This can activate motives that facilitate gender equality (system change motives) while also reducing barriers (system justification motives; Lyubykh et al., 2022). Similarly, fostering climates of procedural justice may encourage managers to not only perceive inequality but also act to address it. These strategies may be especially important when the organization is embedded in broader contexts that are sending conflicting signals.

## A Call to Action

Given the importance of addressing gender inequality in the workplace, we call for scholars to focus on how managers *can* be motivated to perceive and appropriately respond to gender inequality (as well as promote gender equality). We also call for practitioners to consider the importance of cues and motives as well as strategies that can create a shared cue set (e.g., exchanging information) and reduce resistance (e.g., inducing system change rather than system maintenance motives). Together, we encourage scholars and practitioners alike to share cues and activate motives that can address inequality and enhance equality in organizations and beyond.
